# Genomic and metabolomic insights into the biocontrol potential of *Bacillus velezensis* ZHR0 against sugarcane smut

**DOI:** 10.3389/fmicb.2025.1582763

**Published:** 2025-05-13

**Authors:** Kaiyuan Pan, Jun Chen, Huojian Li, Shiqiang Xu, Jihua Wang, Xiaoni Yan, Yueying Zhao, Hongtao Jiang, Abdullah Khan, Muhammad Tahir Khan, Charles A. Powell, Ronghui Wen, Muqing Zhang

**Affiliations:** ^1^Guangxi Key Laboratory of Sugarcane Biology and College of Life Science and Technology, Guangxi University, Nanning, China; ^2^National Institute for Biotechnology and Genetic Engineering (NIBGE), Faisalabad, Pakistan; ^3^IFAS, University of Florida, Fort Pierce, FL, United States

**Keywords:** *Bacillus velezensis*, *Sporisorium scitamineum*, biocontrol, antifungal activity, whole-genome sequencing

## Abstract

Sugarcane smut, caused by *Sporisorium scitamineum*, is a major disease threatening global sugarcane production. Biological control agents (BCAs) offer environmentally sustainable alternatives to chemical fungicides, with *Bacillus velezensis* recognized for its broad-spectrum antifungal properties. In this study, *B. velezensis* ZHR0 was isolated from sugarcane leaves and evaluated for its antifungal activity through *in vitro* dual-culture assays and *in vivo* greenhouse trials. Field application of a ZHR0-based biofertilizer achieved a maximum disease control efficiency of 43.86%. Whole-genome sequencing revealed a 4.04 Mb genome with a GC content of 46.48%, encoding 4,150 genes, including multiple biosynthetic gene clusters (BGCs) associated with secondary metabolite production. *In vitro* assays showed that ZHR0 inhibited the growth of *S. scitamineum* by 53.20% and reduced disease incidence in sugarcane seedlings by 45.74%. Notably, BGCs for iturin, fengycin, surfactin, and difficidin were identified, and liquid chromatography-mass spectrometry (LC-MS) confirmed the production of iturin, supporting its role in antifungal activity. These findings demonstrate the biocontrol potential of *B. velezensis* ZHR0 against sugarcane smut and provide integrated genomic and metabolomic evidence for its application as a sustainable biocontrol agent in sugarcane cultivation.

## 1 Introduction

Sugarcane (*Saccharum* spp. hybrids) is cultivated in over 100 countries, contributing approximately 85% of global sugar production and accounting for over 90% of sugar production in China (Croft et al., 2008; Wang et al., 2023). Sugarcane smut is most devastating and widespread fungal disease caused by *Sporisorium scitamineum* in sugarcane cultivation (Wu et al., 2024). This disease significantly causes reductions in yield and impairs quality across most sugarcane-producing regions (Bhuiyan et al., 2021). Selecting disease-resistant varieties is currently the most effective way to control sugarcane smut. However, the status of sugarcane as an allopolyploid plant with a complex genetic background and an extremely low recombination rate of beneficial genes (about 1/300,000) makes breeding for disease resistance particularly time-consuming (Wu et al., 2022a; Wang et al., 2023). Therefore, it is imperative to develop an effective and environmentally sound biological fungicide.

There has been growing interest in biological control agents (BCAs) as sustainable substitutes for chemical fungicides. BCAs constitute a group of beneficial organisms commonly employed to control the spread and damage caused by pests or pathogens (Lahlali et al., 2022). BCAs are used extensively to biocontrol various plant pathogens in agricultural settings. For instance, *Trichoderma* sp., *Bacillus* spp., *Pseudomonas* spp., and *Burkholderia* spp. effectively inhibit the growth of *Fusarium* spp., *Pythium* spp., *Phytophthora* spp., and *Rhizoctonia solani*, which are responsible for soil-borne and foliar diseases in plants (Compant et al., 2005; Pérez-García et al., 2011). Beyond their inhibitory effects, numerous BCAs have also demonstrated plant growth-promoting properties (Tsalgatidou et al., 2023). *Bacillus* has gained recognition for its dual role in promoting plant growth and controlling pathogens. For instance, *Bacillus velezensis* IFST-221 has exhibited antagonistic activity against eight species of pathogenic ascomycetes and oomycetes, in addition to demonstrating significant plant growth-promoting effects in maize, cotton, tomato, and broccoli seedlings (Liang et al., 2024).

*Bacillus* spp. represents a group of potential endophytic microbes capable of colonizing plant tissues without inflicting harm, making them highly promising candidates for biocontrol strategies against phytopathogens. *Bacillus* spp. produces a series of bioactive secondary metabolites, such as lipopeptides (surfactin, iturin, and fengycin), polyketides (macrolactin, bacillaene, and difficidin), and aminoglycoside (butirosin), which are crucial for inhibiting pathogen infections by changing the structure of cell membranes (Harwood et al., 2018). In addition, *Bacillus* spp. produces glycoside hydrolases, such as chitinase and glucanase, which directly inhibit fungal hyphal growth (Tanaka and Watanabe, 1995). *Bacillus* spp. can also improve plant resistance to disease by inducing systemic resistance (Nguyen et al., 2022). Genome sequencing and bioinformatics analysis enable the identification of secondary metabolite synthesis genes and other gene clusters associated with antifungal activity (Lu et al., 2022; Thiruvengadam et al., 2022). This approach provides a significant understanding of the molecular mechanisms underlying *Bacillus*-mediated biological control and its role in promoting plant growth.

Although *Bacillus* spp. has shown efficient biocontrol ability in various crops, limited research has focused on the control and application of *B. velezensis* for sugarcane smut. Given the deleterious impact of *S. scitamineum*, finding an environmentally sustainable approach to disease management is paramount. In this study, *B. velezensis* ZHR0, an endophyte isolated from sugarcane leaves, was identified as a member of the *Bacillus* genus with biocontrol potential similar to previously reported strains. The strain was assayed for antifungal activity against *S. scitamineum* and demonstrated broad-spectrum antifungal activity against various phytopathogenic fungi. Further, the biological control efficacy of ZHR0 on sugarcane smut was studied in a pot and field experiment. The whole genome sequencing of ZHR0 and comparative genomic analyses were conducted to ascertain its secondary metabolism gene clusters and genes involved in plant probiotic functions. In addition, the crude extract of ZHR0 was subjected to antimicrobial activity assessment and characterization. Our research provides an environmentally friendly and efficient solution for controlling sugarcane smut.

## 2 Materials and methods

### 2.1 Isolation and culture of ZHR0 and pathogenic

The strain of *B. velezensis* ZHR0 was isolated from sugarcane leaves collected from a sugarcane smut-affected area at the Guangxi University Research Station in Fusui, Chongzuo, China (22°38′06″“N, 107°54′15”″ E). Leaf samples (10 g) were surface sterilized by immersing them in 0.1% HgCl_2_ for 30 s, followed by 75% ethanol for 2 min, and rinsing three times with sterile water. The sterilized leaf pieces were mixed with 100 mL sterile water and shaken at 37°C for 30 min. Stepwise dilutions (10^−1^, 10^−2^, 10^−3^, 10^−4^, and 10^−5^) were prepared, and 100 μL of each dilution was spread on Luria-Bertani (LB) agar plates and incubated at 37°C for 2 days. After incubation, a pure colony was obtained.

The ZHR0 strain was also cultured in LB shaken at 210 rpm for 48 h at 37°C in the dark to obtain a fresh bacterial suspension. The strain ZHR0 is currently preserved at the China Center for Type Culture Collection (CCTCC) under accession number CCTCC NO: M 2014577. *Sporisorium scitamineum*, used in this study, was isolated from sugarcane field samples and identified in our laboratory. The fungal strains were cultured on potato dextrose agar (PDA) and incubated at 28°C in the dark with shaking at 210 rpm for 48 h. For co-culture experiments, 5 μL of ZHR0 bacterial suspension was inoculated at the center of a PDA plate. Subsequently, 5 μL of *S. scitamineum* suspension (1 × 10^6^ CFU/mL) was inoculated 2 cm away from the center along two perpendicular lines. The plates were incubated for 3 days at 28°C.

### 2.2 Identification and genomic analysis of ZHR0

Colony morphology was observed on LB agar plates, including color, shape, and surface texture. For molecular characterization, the 16S rRNA gene fragment of ZHR0 was amplified using polymerase chain reaction (PCR). The reaction was prepared in a 50 μL final volume containing 25 μL Taq Master Mix (Vazyme, China), 1 μL forward primer, 1 μL reverse primer, 1 μL bacterial culture, and 22 μL double-distilled water (ddH2O). The PCR conditions included an initial denaturation step at 95°C for 4 min, followed by 35 cycles of 95°C for 10 s, 55°C for 30 s, and 72°C for 2 min. The amplified products were sequenced by Sangon Biotech Co., Ltd. (Shanghai, China). The resulting 16S rRNA sequences were used for phylogenetic analysis, which was performed using MEGA7 software, following the approach described by Gorai et al. (2021).

The genome of the ZHR0 strain was sequenced and spliced using the PacBio sequencing platform, employing third-generation sequencing technology. The genome was assembled using the overlap-layout-consensus (OLC) algorithm implemented in HGAP within the SMRT Analysis software (v2.3.0). Circularization of the ZHR0 genome was completed using Circos software (v0.69). Coding genes were predicted from the assembled genome using Glimmer software. Functional annotation of the predicted genes was performed using six major databases: NR, NT, Swiss-Prot, COG, Kyoto Encyclopedia of Genes and Genomes (KEGG), and Gene Ontology (GO).

The whole genome sequences of *B. velezensis* FZB42 (NC_009725.2), SQR9 (NZ_CP006890.1), J17-4 (NZ_CP060420.1), *B. amyloliquefaciens* DSM7 (NC_014551.1), *B. subtilis 168* (NC_000964.3), *B. siamensis* JJC33M (NZ_JTJG01000001.1), and *B. thuringiensis* BT62 (NZ_CP044978.1) were downloaded from the NCBI. The average nucleotide identity was calculated using the JSpecies web server^1^ and visualized with ChiPlot.^2^ Comparative analysis of orthologous genes among the protein sequences of *B. velezensis* ZHR0, FZB42, SQR9, *B. amyloliquefaciens* DSM7, and *B. subtilis* 168 was performed using OrthoVenn 3.^3^

### 2.3 Detection of broad-spectrum antifungal ability of ZHR0

The broad-spectrum antifungal activity of *B. velezensis* ZHR0 was evaluated using a dual culture method. Mycelial plugs (6 mm) were placed at the center of 90 mm PDA plates. A ZHR0 bacterial solution with an OD_600_ = 1 was inoculated at four equidistant points 2 cm away from the mycelial plug (10 μL per point). As a control, 10 μL of LB was inoculated at the corresponding points on separate plates. All plates were incubated at 28°C until the fungal colonies on the control plates reached full growth. The inhibition rate (%) was calculated as:


$$\text{Inhibition rate}\;(\%)=\frac{(D1-D2)\;}{D2}\times 100$$

Where D1 represented the fungal colony diameter (mm) on the control plate, and D2 represented the fungal colony diameter (mm) on the ZHR0-inoculated plate. The fungal pathogens *Sclerotium rolfsii* Sacc, *Colletotrichum asianum*, *Colletotrichum gloeosporioides*, and *Botrytis cinerea* were provided by the Plant Pathology Teaching and Research Group, College of Agriculture, Guangxi University. *Fusarium commune* was isolated from field samples and maintained in our laboratory.

### 2.4 Construction of ZHR0-GFP

The plasmid PCM20, an *Escherichia coli*-*Bacillus subtilis* shuttle vector containing the GFP gene and the erythromycin resistance gene, was provided by Dr. Bo Liu (Agricultural Bio-Resource Institute, Fujian Academy of Agricultural Sciences). The PCM20 plasmid was introduced into ZHR0 using a modified high-osmolality electroporation protocol (Xue et al., 1999). Specifically, ZHR0 was cultured in 50 mL LB (containing 0.5 M sorbitol) at 37°C with shaking until the OD_600_ reached 0.6~0.8. Cells were harvested by centrifugation and washed three times with an ice-cold electroporation medium (0.5 M sorbitol, 0.5 M mannitol, and 10% glycerol). The pellet was resuspended in 1/50 of the original culture volume using the same electroporation medium. For electroporation, 100 μL of prepared cells were mixed with 1 μL of plasmid DNA (50 ng/μL) and transferred to an ice-cold electroporation cuvette (2 mm electrode gap). The mixture was incubated on ice for 5 min before exposure to an electrical pulse using a Gene-Pulser (Bio-Rad Laboratories, Richmond, CA) set at 25 mF and 1.8 kV, resulting in a time constant of 4.5–5.0 ms. Immediately after electroporation, 1 mL LB medium was added to the cuvette, and the mixture was transferred to a 2 mL EP tube. After incubation at 30°C for 1 h, the cells were plated on LB-agar containing 3 μg mL-1 erythromycin to select transformants.

### 2.5 Extraction and detection of ZHR0 crude extracts

The extraction and assay of ZHR0 crude extracts were based on the method described by Grover et al. (2010), with modifications. ZHR0 was cultured on LB medium for 144 h at 37°C with shaking at 220 rpm. The cell-free supernatant (CFS) was collected by centrifugation (8,000 rpm for 25 min) and filtration through a sterile 0.22-μm filter membrane. The pH of CFS was adjusted to 2.0 using HCl (6 mol/L), and the acid-precipitated material was recovered by centrifugation at 10,000 rpm for 15 min at 4°C. The precipitate was then extracted with methanol, and the resulting methanol extract was dried under vacuum to yield a light brown residue, representing the crude extract of ZHR0. The dried crude extract was dissolved in methanol (HPLC-grade) and filtered through a sterile 0.22 μm filter membrane to obtain the purified methanol crude extract (ME). The extract was adjusted to a 100 mg/mL concentration and incorporated into a PDA medium at a concentration of 1% (v/v) for antifungal assays.

The samples were processed for chemical analysis using an ultra-high-performance liquid chromatography (UHPLC) system (Shimadzu—lc30, Shimadzu Co., Ltd., China). Chromatographic separation was performed on a Waters Corporation (Milford, MA, United States) ACQUITY UPLC® HSS T3 column (2.1 × 100 mm, 1.8 μm particle size). After UHPLC separation, mass spectrometry analysis was conducted using a QE Plus mass spectrometer equipped with a heated electrospray (HESI) ionization source.

### 2.6 Anti-smut activity assays *in planta*

*Bacillus velezensis* ZHR0 was inoculated into LB medium and incubated in a shaker at 37°C for 48 h. The fermentation broth was centrifuged at 5,000 rpm for 20 min, and after removing the supernatant, it was rinsed with sterile water and centrifuged again at 5,000 rpm. The rinsing was repeated three times, and the ZHR0 was adjusted to 2 × 10^8^ cfu/mL with sterile water. After inoculating *S. scitamineum* spores into PDA at 28°C for 48 h, the same rinsing was done three times, and the spores were adjusted to 1 × 10^6^ cfu/mL.

The healthy cane stalk from ROC22, susceptible to sugarcane smut, was cut into single bud segments, soaked in water for 1 h, and incubated in a constant temperature incubator at 30°C. After 3 days, uniformly growing seedlings were selected for inoculation. The ZHR0 suspension was mixed with *S. scitamineum* suspension in equal volume, and 5 μL of the mixture was inoculated into sugarcane buds using a syringe. Sterile water mixed with *S. scitamineum* suspension in equal volume was used as inoculation as a control. Each treatment was inoculated with 30 buds in three replications. The control and treated canes were incubated separately in a 28°C incubator and transferred to continue incubation in the greenhouse after 24 h. The germination rate and incidence rate of sugarcane were recorded. The disease incidence of smut was quantified by calculating the percentage of infected plants relative to the total plant population.


$$\text{Incidence}\;(\%)=\frac{\text{No}.\text{of diseased plants}}{\text{Total}\;\text{no}.\text{of plants investigated}}\times 100$$

$$\begin{array}{l} \text{Biocontrol effect efficiency}\;(\%)=\\ \frac{\text{control incidence}-\text{treatment incidence}}{\text{control incidence}}\times 100 \end{array}$$

### 2.7 Field evaluation of sugarcane smut disease

Field experiments were conducted at the Fusui Agricultural Experimental Station of Guangxi University, Fusui, China (22°51′N, 107°78′E), using the sugarcane variety GT42. Nine blocks were set up for three treatments, each with rows of 15 m in length. The basic compost contained sugarcane bagasse (0.5~1 cm), spent mushroom substrate, and an organic starter (JunDe Biotechnology Co., Ltd. Shandong, China). The basic compost preparation involved mixing the spent mushroom substrate with bagasse in a 1:1 mass ratio, adjusting the C/N ratio to 30:1 using urea, inoculating with a fermentation agent, and maintaining the moisture content of the compost at about 65%. The basic compost was then mixed with ZHR0 bacterial suspension at a mass-to-volume ratio of 7:3 (kg ·L ^−1^). After fermentation, once ZHR0 had successfully colonized the compost and reached a density of 10^6^ CFU/g, the material was used as a ZHR0 biofertilizer for the experiments. The experimental treatments were as follows; (1) CK = basic compost (3,000 kg·ha^−1^), (2) BF1 = basic compost (1,500 kg·ha^−1^) + ZHR0-biofertilizer (1,500 kg·ha^−1^), (3) BF2 = ZHR0-biofertilizer (3,000 kg·ha^−1^). Disease was investigated 6 months after sugarcane emergence. The total number of plants and the total number of diseased plants were recorded for each replicate of each treatment.

### 2.8 Characterization of extracellular enzymatic activities and plant growth-promoting traits of *Bacillus velezensis* ZHR0

To evaluate the plant growth-promoting and extracellular enzymatic capabilities of strain ZHR0, a series of qualitative plate-based assays were conducted.

Protease activity was assessed using skim milk agar plates, prepared by dissolving 12 g skim milk powder and 20 g agar in 1,000 mL of double-distilled water (ddH_₂_O), followed by autoclaving.

Cellulase activity was evaluated using carboxymethyl cellulose (CMC) agar medium consisting of 10 g sodium CMC, 10 g peptone, 20 g agar, 5 g yeast extract, 1 g KH_₂_PO_₄_, and 5 g NaCl in 1,000 mL ddH_₂_O, adjusted to pH 7.0.

Phosphate solubilization ability was determined on inorganic phosphate medium containing 10.0 g glucose, 0.30 g NaCl, 0.30 g KCl, 0.3 g MgSO_₄_·7H_₂_O, 0.50 g (NH₄)₂SO₄, 20 g agar, 0.03 g MnSO_₄_·4H_₂_O, 5.0 g MgCl_₂_, 0.03 g FeSO_₄_·7H_₂_O, and 5.0 g Ca_₃_(PO_₄_)_₂_ per 1,000 mL ddH_₂_O, with the pH adjusted to 7.0–7.5 prior to sterilization.

Amylase activity was tested using starch hydrolysis medium containing 5 g/L yeast extract, 10 g/L peptone, 5 g/L NaCl, 30 g/L soluble starch, and 15 g/L agar, adjusted to pH 7.6.

For indole-3-acetic acid (IAA) production, ZHR0 was inoculated into Luria-Bertani (LB) broth supplemented with 0.2 g/L L-tryptophan and incubated at 37°C for 48 h with shaking at 180 rpm. Following incubation, cultures were centrifuged to obtain cell-free supernatant. Equal volumes of the supernatant and Salkowski reagent (prepared by dissolving 150 mg FeCl_₃_·6H_₂_O in 1 L of concentrated H_₂_SO_₄_) were mixed at a 1:1 (v/v) ratio. The mixture was incubated in the dark for 30 min, and absorbance was measured at 535 nm using a UV–Visible spectrophotometer.

### 2.9 Statistical analysis

All experiments were conducted in triplicate. Statistical analyses were performed using SPSS 26.0 (IBM Corp., USA). One-way analysis of variance (ANOVA) followed by Tukey’s HSD test was used to compare group means. Results are expressed as mean ± standard deviation. Differences were considered statistically significant at *p* < 0.05.

## 3 Results

### 3.1 Identification of strain ZHR0 with broad-spectrum antifungal activity

**Figure 1:**
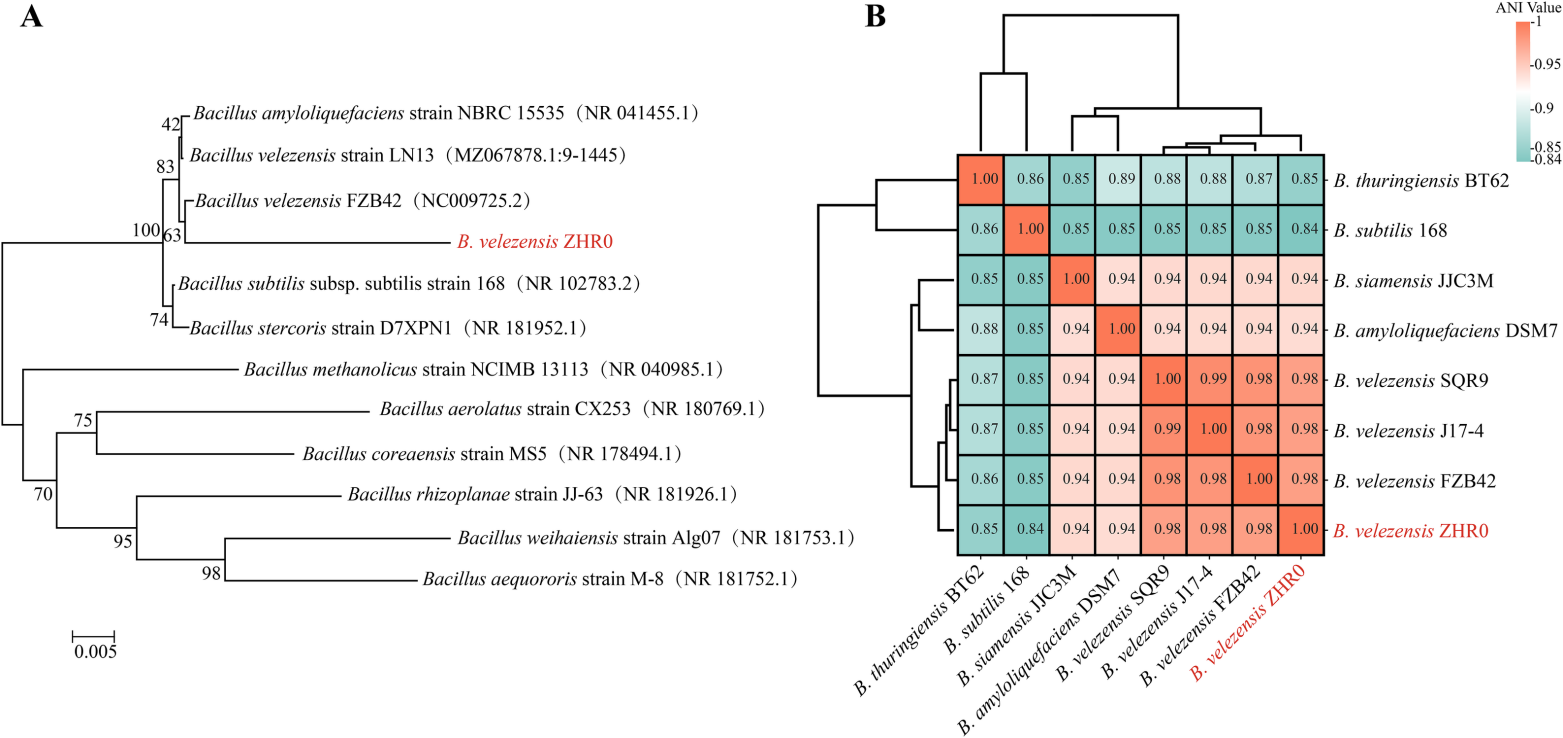
Molecular characterization and genomic comparison of strain ZHR0. **(A)** Phylogenetic tree of ZHR0 based on 16S rRNA sequences. **(B)** Heatmap generated from the ANI values by comparing the genomes of strain ZHR0 to the other strains.

Upon observation, the surface of strain ZHR0 appeared milky white with irregular folds at the edges (Supplementary Figure S1). We performed PCR amplification and sequencing of the 16S rRNA gene to determine its taxonomic status. Phylogenetic analysis based on the 16S rRNA gene sequence placed strain ZHR0 within the same clade as *B. velezensis* (Figure 1A). Based on the reclassification of *Bacillus velezensis* and *Bacillus amyloliquefaciens* in 2016 (Dunlap et al., 2016), we further confirmed the identity of ZHR0 using whole-genome sequencing. The average nucleotide identity (ANI) analysis revealed that strain ZHR0 showed the highest similarity (>98%) to *B. velezensis* strains FZB42, SQR9, and J17-4 (Figure 1B). Based on these findings, we identified strain ZHR0 as *B. velezensis*.

**Figure 2:**
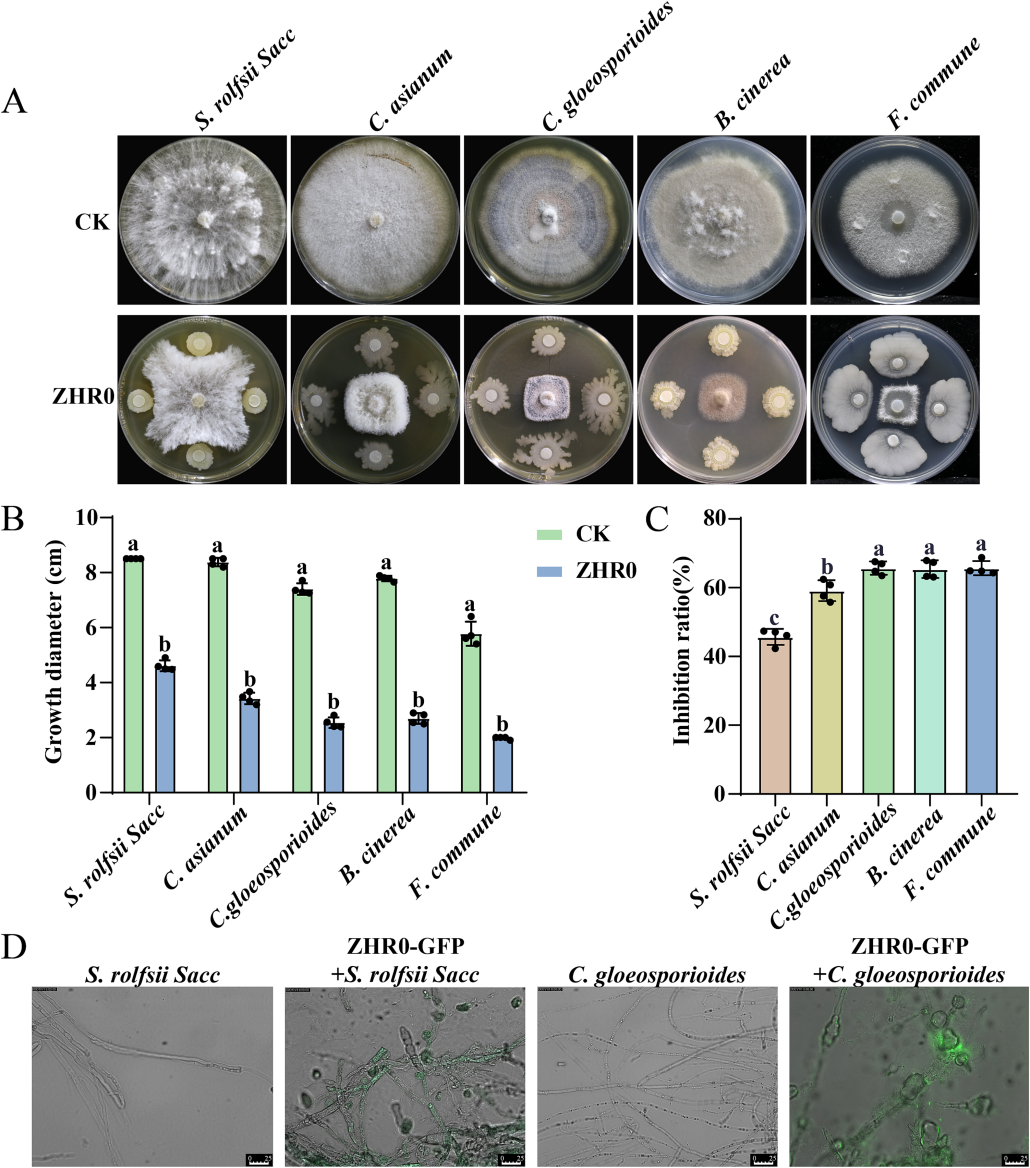
Broad-spectrum antifungal ability of ZHR0. **(A)** Inhibitory activity of ZHR0 against different pathogenic fungi *in vitro*. *Sclerotium rolfsii* Sacc, *Colletotrichum asianum*, *Colletotrichum gloeosporioides*, *Botrytis cinerea*, *Fusarium commune* are used for antifugal activity. **(B)** Growth diameter of ZHR0 against different phytopathogens. Error bars represent standard errors. Different lowercase letters indicated a significant difference between the two treatments according to the Student’s t-test (*p* < 0.05). **(C)** Inhibition rates of *Bacillus velezensis* ZHR0 against five phytopathogens. Error bars represent standard errors. Different lowercase letters indicated a significant difference among different treatments according to the one-way ANOVA (*p* < 0.05). **(D)** Effect of ZHR0-GFP on mycelium of *S. rolfsii* Sacc, *C. gloeosporioides* by fluorescence microscopy, bar = 25 μm.

The broad-spectrum antifungal activity of *B. velezensis* ZHR0 was assessed against various phytopathogens including *S. rolfsii* Sacc, *C. asianum*, *C. gloeosporioides*, *B. cinerea*, and *F. commune*. The results revealed that ZHR0 had robust antagonistic ability against all tested pathogens, including deuteromycetes and ascomycetes (Figure 2A). The phytopathogen colony diameter was significantly smaller than the control group (Figure 2B). The lowest inhibition rate was observed against *S. rolfsii* Sacc (45.71%), whereas the highest inhibition rates, exceeding 60%, were achieved for *C. gloeosporioides*, *B. cinerea*, and *F. commune* (Figure 2C). A fluorescence microscope (IMAGER Z1) was employed to examine the ultrastructural effect of ZHR0-GFP on fungal hyphae, focusing on strains such as *S. rolfsii* Sacc and *C. gloeosporioides*. Images of untreated *S. rolfsii* Sacc hyphae displayed smooth surfaces and intact hyphae. In contrast, hyphae treated with ZHR0-GFP showed obvious morphological changes, including folding, twisting, and partial expansion (Figure 2D). Similarly, irregular hyphal structures were observed in *C. gloeosporioides* treated with ZHR0-GFP (Figure 2D). These findings suggested that ZHR0 treatment induced morphological alterations in the hyphae of the tested pathogens.

### 3.2 Antagonistic activity *of Bacillus velezensis* ZHR0 against *Sporisorium scitamineum in vitro* and *planta*

**Figure 3:**
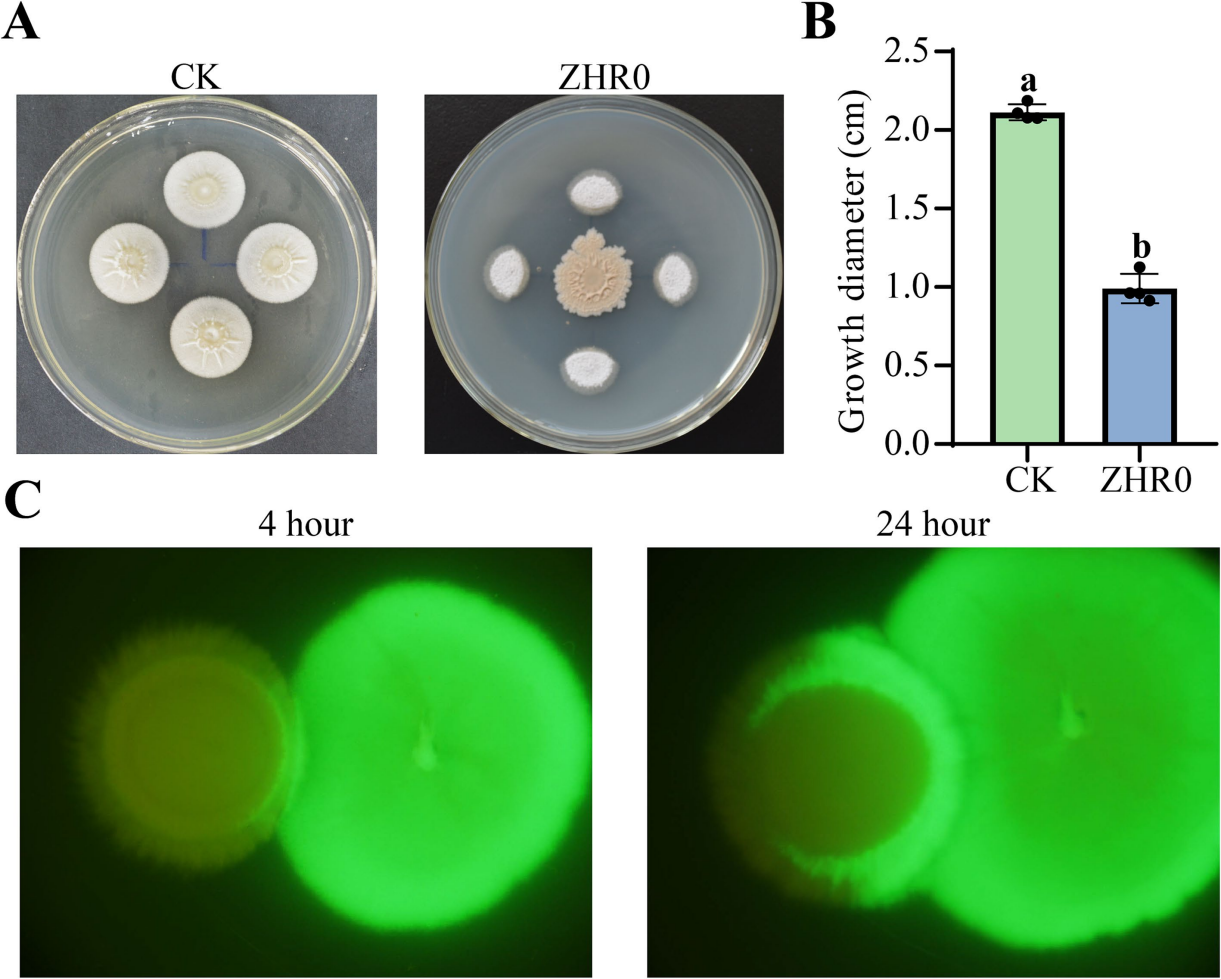
Antifungal activity of ZHR0 against *Sporisorium scitamineum*. **(A,B)** Inhibitory capacity of ZHR0 against *S. scitamineum in vitro*. Error bars represent standard errors. Different lowercase letters indicated a significant difference between the two treatments according to the Student’s t-test (*p* < 0.05). **(C)** Infestation of *S. scitamineum* by ZHR0-GFP at 4 h, 24 h.

A dual-culture assay was conducted to determine the antifungal activity of ZHR0 against the sugarcane smut fungus *S. scitamineum*. In a dual-culture assay, the growth of *S. scitamineum* near the ZHR0 side was significantly inhibited. In contrast, its growth away from ZHR0 proceeded faster, resulting in abnormal colony shapes compared to the control (Figure 3A). Strain ZHR0 showed significant antifungal ability against *S. scitamineum*, with an inhibition rate of up to 53.20% (Figure 3B). The interaction between strain ZHR0 and *S. scitamineum* was also visualized using a GFP-labeled strain. Green fluorescence was observed in *S. scitamineum* over time when co-cultured with ZHR0-GFP, confirming the ability of the strain to infest the pathogen (Figure 3C).

**Figure 4:**
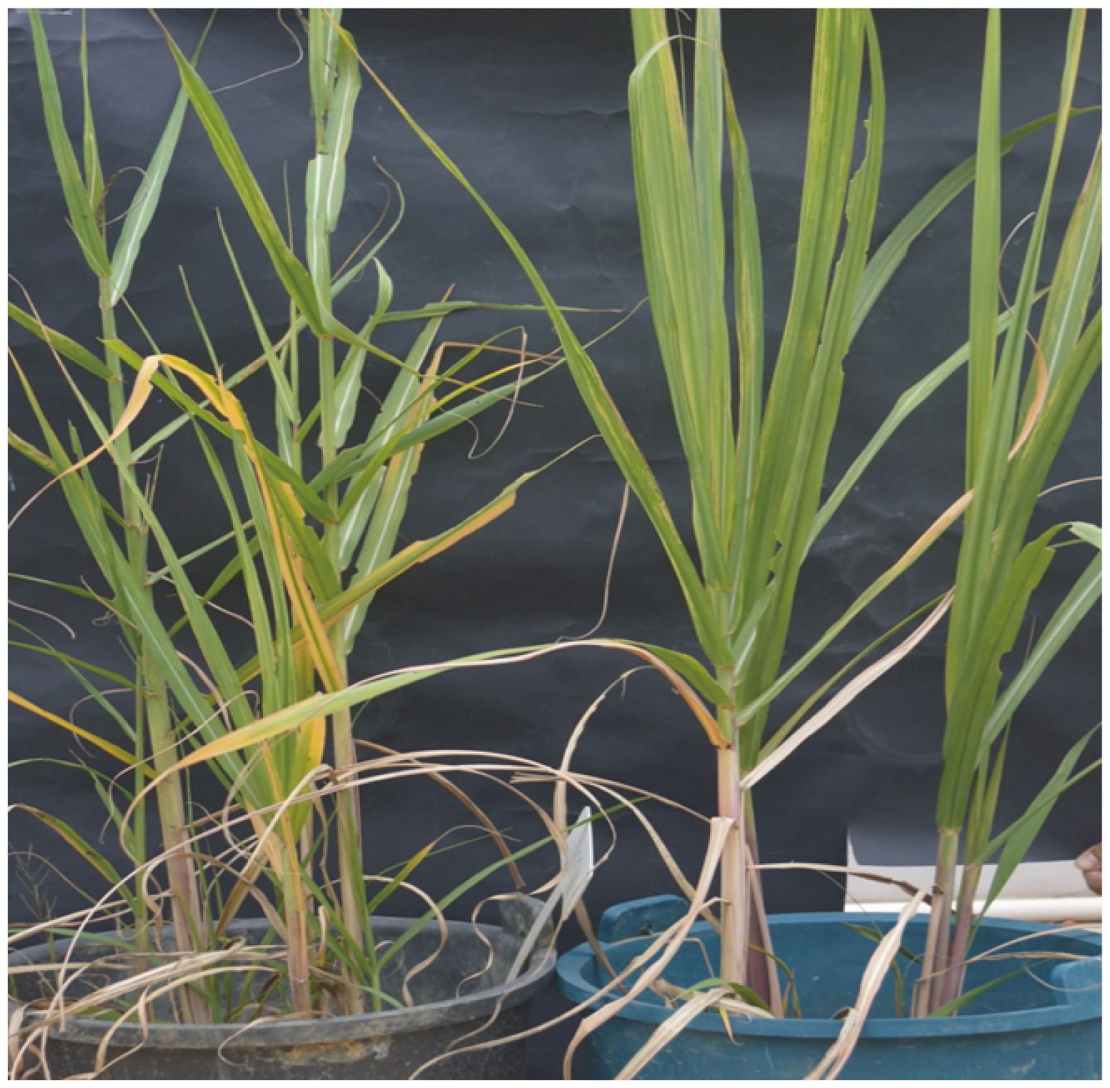
Antifungal activity of *Bacillus velezensis* ZHR0 in suppressing sugarcane smut under potted conditions. The black bucket on the left is the control and the blue bucket on the right is the ZHR0 treatment. Control (left, black bucket) and ZHR0-treatment (right, blue bucket) groups are illustrated in the figure.

Antifungal activity was also assessed using a sugarcane seedling assay to evaluate further the biocontrol efficacy of *B. velezensis* ZHR0 against sugarcane smut *in planta*. The seedling emergence rates were comparable between the control (88.89%) and ZHR0-treatment groups (87.78%). Disease symptoms, characterized by the development of black whips, became evident 93 days post-inoculation with daily observation and immediate removal of symptomatic stems to prevent secondary infection. After 60 days, the number of diseased plants in the control and treatment groups was counted, and the incidence rate was calculated. The incidence rate of the control group was 86.25%, and ZHR0 treatment was 46.84%. The control efficiency reached 45.69% (Table 1; Figure 4). The above results indicated that the ZHR0 strain had a significant effect against sugarcane smut *in planta*.

**Table 1:** Incidence and control efficiency of sugarcane smut in ZHR0-treatment.

Treatment	Seedling rate	Incidence	Biocontrol efficiency (%)
CK	88.89 ± 1.92	86.23 ± 2.32	/
ZHR0-treament	87.78 ± 1.92	46.81 ± 4.01	45.74 ± 3.67

Data are presented as mean ± SD.

A field experiment conducted 6 months post-application assessed the impact of different ZHR0 biofertilizer treatments on sugarcane smut control. Sugarcane plants pre-treated with ZHR0 biofertilizer showed significantly lower smut incidence rates (6.57% and 6.05%) compared to untreated plants (10.7%) (Table 2). The smut control efficiency of the biofertilizer treatments, BF1 and BF2, was as high as 38.97% and 43.86%, respectively. These results further indicated that ZHR0 biofertilizer effectively reduces smut incidence in field conditions.

**Table 2:** Incidence and control efficiency of sugarcane smut in different treatments.

Treatment	Incidence (%)	Control efficiency (%)
CK	10.7 ± 0.7a	/
BF1	6.57 ± 1.25b	38.97
BF2	6.05 ± 1.47b	43.86

Data are presented as mean ± SD. Different lowercase letters indicated a significant difference among different treatments according to the one-way ANOVA (*p* < 0.05).

### 3.3 Genomic feature of *Bacillus velezensis* ZHR0

**Figure 5:**
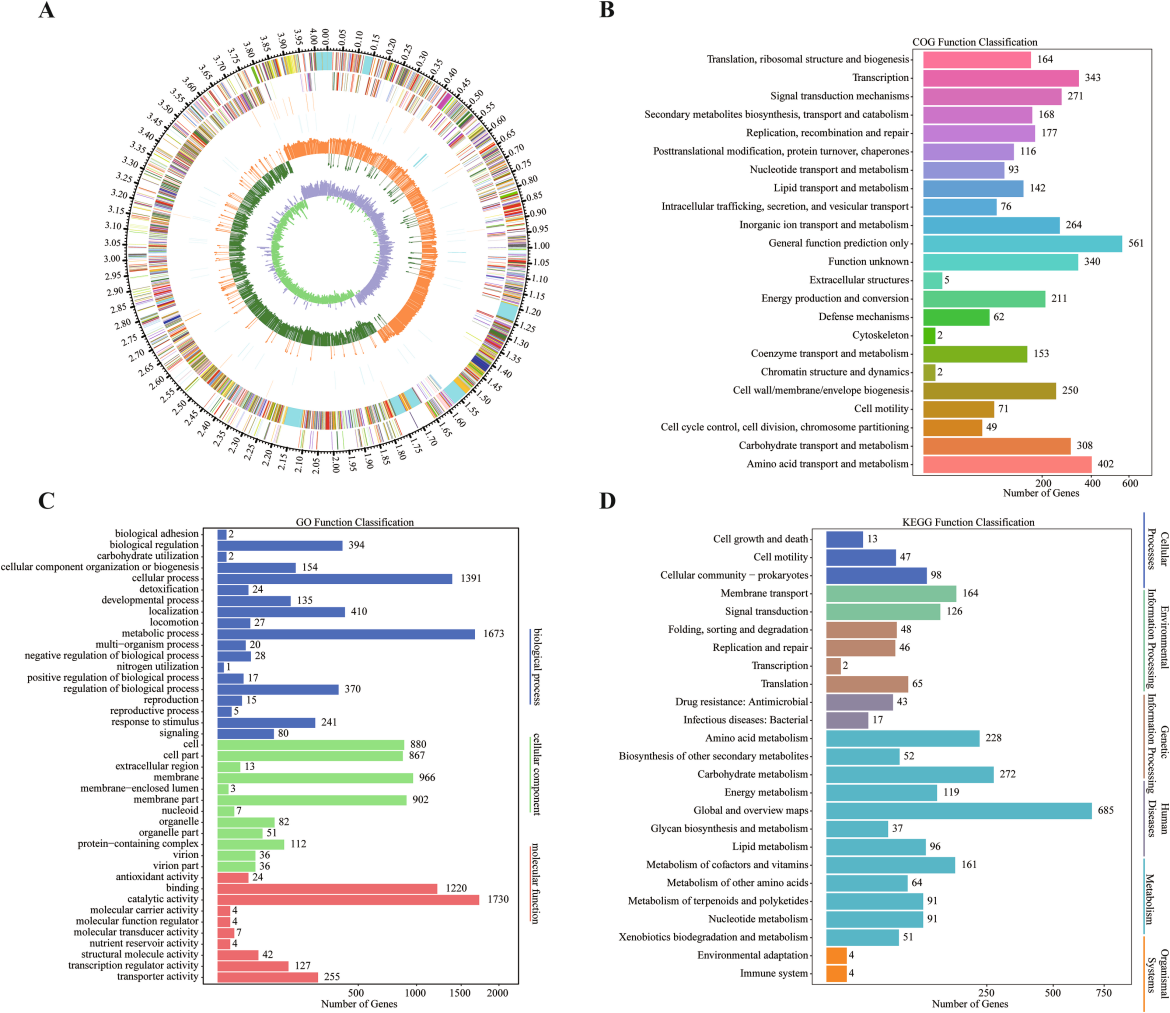
Genomic information and gene annotation for *Bacillus velezensis* ZHR0. **(A)** Circular genomic map of ZHR0. The first circle shows the genome from the outside to the inside (the scale is the specific position). The second circle shows the distribution of COG-annotated genes with positive strands, distinguished by different colors. The third circle indicates the distribution of COG-annotated genes with negative strands. The fourth circle shows the distribution of tRNAs. The fifth circle shows the distribution of repetitive sequences. The sixth circle shows the GC contents of positive-stranded genes. The seventh circle shows the GC contents of negative-stranded genes. The eighth circle shows the GC skew of positive-stranded genes; the ninth circle shows the GC skew of negative-stranded genes. **(B)** Annotation information of ZHR0 genes in COG database. **(C)** Annotation information of ZHR0 genes in GO database. **(D)** Annotation information of ZHR0 genes in KEGG database.

Whole genome sequencing of *B. velezensis* ZHR0 yielded significant insights into its genomic features. Following sequencing and quality assessment of the original reads, the assembled genome consisted of a circular chromosome with a length of 4,040,268 bp and a GC content of 46.48% (Figure 5A; Table 3). A total of 4,150 genes were identified within the genome, with an average gene length of 865 bp, representing approximately 88.88% of the total genome length, with a combined gene length of 3,590,886 bp (Table 3). Additionally, the genome analysis predicted the presence of 86 tRNA structures, 26 rRNA operons (comprising 5S, 16S, and 23S structures), and 14 biosynthetic gene clusters (BGC) (Table 3).

**Table 3:** Genomes features of *Bacillus velezensis* ZHR0.

Strain name	ZHR0
Genome size (bp)	4,040,268
GC content (%)	46.48
Number of coding genes	4,150
Average CDS length	865
Gene total Length (bp)	3,590,886
Gene Length/ Genome (%)	88.88
tRNA number	86
rRNA number	26
Cluster number	14

The majority of *B. velezensis* ZHR0 genes were annotated using non-redundant nucleotide and protein sequences from the NT, NR, Swiss-Prot, COG, Gene Ontology (GO), and Kyoto Encyclopedia of Genes and Genomes (KEGG) databases, with annotation rates of 99.57% (4,132), 99.47% (4,128), 87.13% (3,616), 72.41% (3,005), 71.20% (2,955), and 52.43% (2,176), respectively (Supplementary Table S1). Of the 2,955 genes annotated in the COG database and classified into 23 categories, the largest proportion was annotated to the “general function prediction only” category (561 genes), followed by “amino acid transport and metabolism” (402 genes) and “transcription” (343 genes) (Figure 5B). The annotated genes in the GO database were divided into three categories, with the largest number falling under the “biological process” category, followed by the “cellular component” and “molecular function” categories (Figure 5C). A total of 2,176 genes were assigned to six KEGG pathways, which were further distributed across 25 categories. The category with the highest prevalence was “metabolism” (1,947, 89.48%), followed by “environmental information processing” (301, 13.83%) and “genetic information processing” (161, 7.40%) (Figure 5D).

### 3.4 Biosynthetic gene clusters and comparative genomic analysis

**Figure 6:**
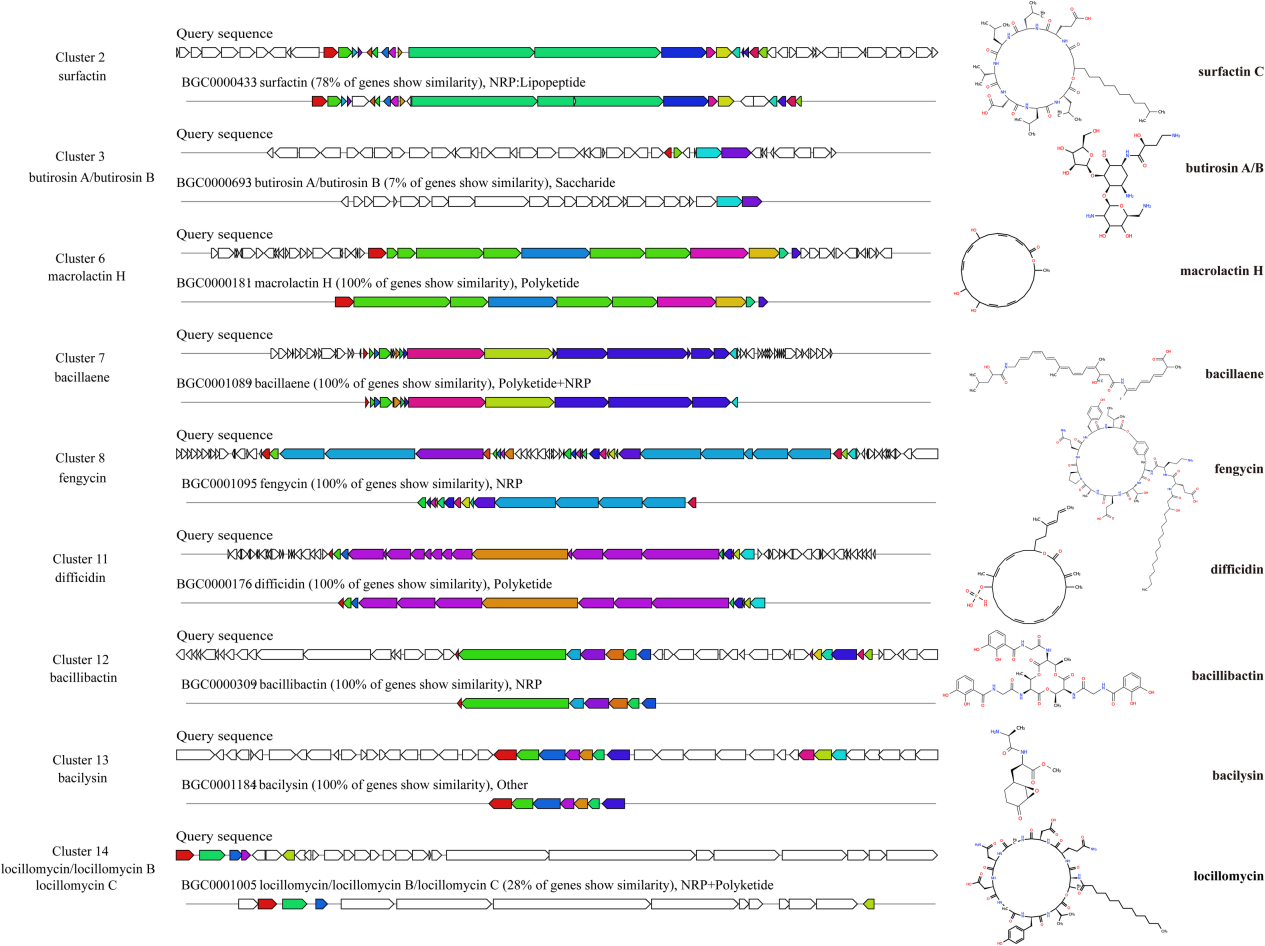
Predicted biosynthetic gene clusters and their associated chemical structures of ZHR0. Left section displays BGCs that share similarities with known gene clusters identified by antiSMASH v7.1.0. Right section shows the chemical structure of a representative BGCs product.

The BGCs in the genome of ZHR0 were successfully identified using the online tool antiSMASH. A total of 14 BGCs were annotated and categorized into 10 categories, including NRPS, transAT-PKS-like, PKS-like, terpene, lanthipeptide-class-ii, transAT-PKS, betalactone, T3PKS, NRP-metallophore, and RiPP-like (Table 4). Among these, nine BGCs showed high similarity to known gene clusters, including those responsible for producing surfactin, butirosin A/B, macrolactin H, bacillaene, fengycin, difficidin, bacillibactin, bacilysin, and locillomycinA/B/C (Figure 6). Six clusters exhibited 100% similarity with macrolactin H, bacillaene, fengycin, difficidin, bacillibactin, and bacilysin (Figure 6). Clusters 1, 4, 5, 9, and 10 did not match any similar gene clusters, and two BGCs displayed less than 50% similarity, indicating that these clusters might be related to the biosynthesis of unique, undiscovered metabolites (Table 4). In addition to the known secondary metabolites mentioned above, other secondary metabolites were also identified within the BGCs of ZHR0, such as plipastatin, bacillomycin D, mycosubtilin, iturin, and other antimicrobial substances, excluding fengycin, in the known clusters compared in cluster 8 (Supplementary Figure S2).

**Table 4:** Prediction and functional annotation of secondary metabolites of *Bacillus velezensis* ZHR0.

Cluster	Type	From	To	Most similar known cluster	Similarity	MIBiG BGC-IDa
Cluster 1	NRPS, transAT-PKS-like	1	34,457	Unknown		
Cluster 2	NRPS	103,752	169,159	Surfactin (NRP: Lipopeptide)	78%	BGC0000433
Cluster 3	PKS-like	699,051	740,295	Butirosin A/butirosin B (Saccharide)	7%	BGC0000693
Cluster 4	terpene	822,316	843,056	Unknown		
Cluster 5	lanthipeptide-class-ii	977,910	1,006,799	Unknown		
Cluster 6	transAT-PKS	1,170,768	1,258,980	Macrolactin H (Polyketide)	100%	BGC0000181
Cluster 7	transAT-PKS, NRPS	1,482,384	1,592,503	Bacillaene (Polyketide + NRP)	100%	BGC0001089
Cluster 8	NRPS, transAT-PKS, betalactone	1,647,092	1,784,915	Fengycin (NRP)	100%	BGC0001095
Cluster 9	terpene	1,807,470	1,829,353	Unknown		
Cluster 10	T3PKS	1,897,981	1,939,081	Unknown		
Cluster 11	transAT-PKS	2,098,003	2,204,162	Difficidin (Polyketide)	100%	BGC0000176
Cluster 12	NRP-metallophore, NRPS, RiPP-like	2,814,978	2,866,770	Bacillibactin (NRP)	100%	BGC0000309
Cluster 13	other	3,463,361	3,504,779	Bacilysin (Other)	100%	BGC0001184
Cluster 14	NRPS, transAT-PKS	3,983,969	4,040,268	Locillomycin/locillomycin B/locillomycin C (NRP + Polyketide)	28%	BGC0001005

**Figure 7:**
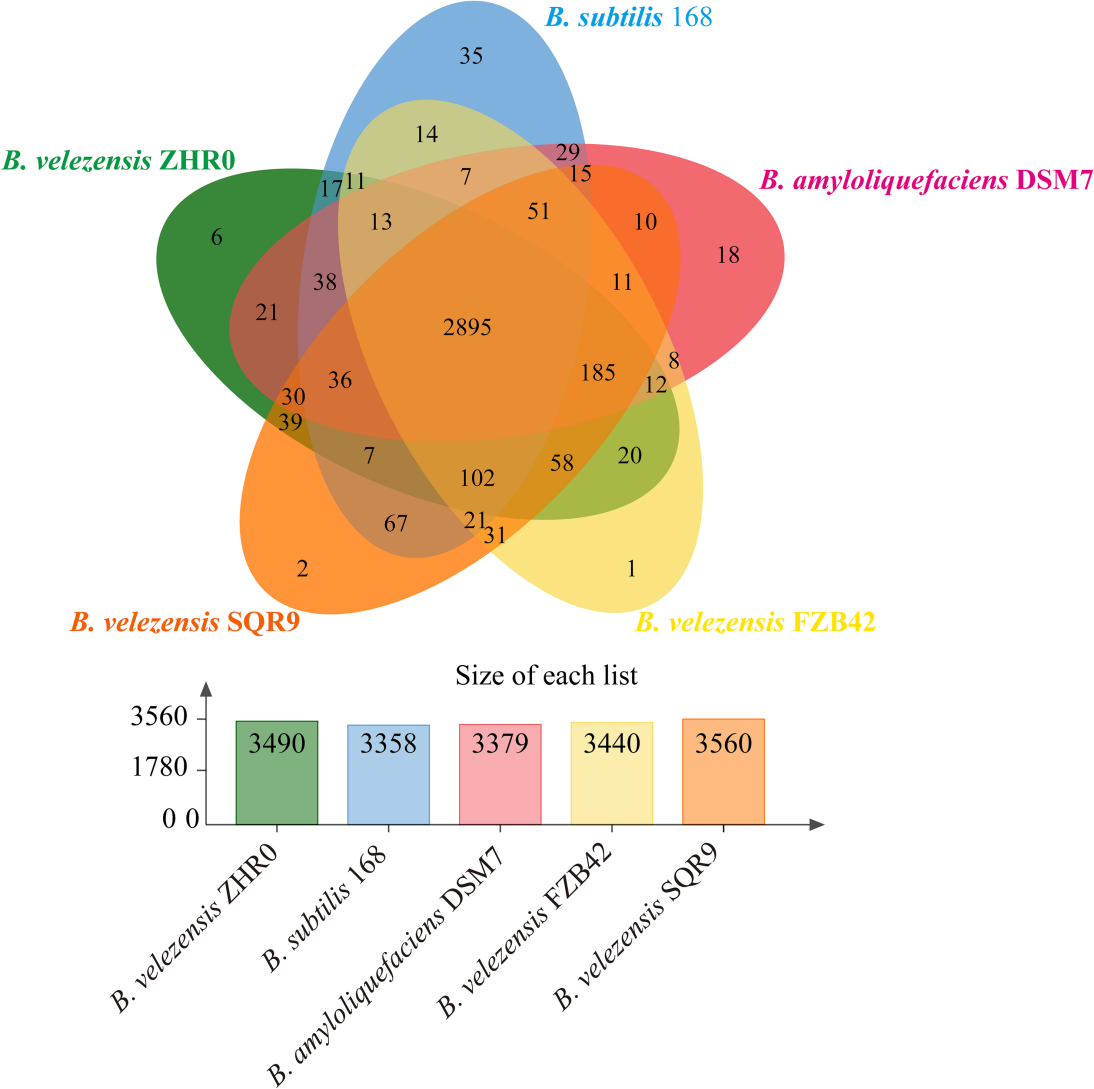
Orthologous analysis and comparison between ZHR0 with other *Bacillus* proteins. Venn diagram display the number of unique, accessory, and core genes shared by *Bacillus velezensis* ZHR0, SQR9, FZB42, *Bacillus amyloliquefaciens* DSM7, and *Bacillus subtilis* 168. The bar graph comparison shows the number of orthologous clusters in each *Bacillus*.

To further characterize the ZHR0 genome, comparative analysis was performed using the genomes of *B. velezensis* FZB42 (Chen et al., 2007), *B. velezensis* SQR9 (Zhang et al., 2015), *B. amyloliquefaciens* DSM7 (Rückert et al., 2011), and *B. subtilis* 168 (Kunst et al., 1997). The protein sequences of the five *Bacillus* strains were compared for orthologous and paralogous gene clusters using OrthoVenn3. The results indicated that out of the 4,150 proteins identified in ZHR0, 3,490 orthologous and paralogous gene clusters were shared across the five *Bacillus* strains (Figure 7). In addition, 2,895 core orthologous gene clusters were common in all five strains, suggesting a high degree of genetic overlap between ZHR0 and the other four strains (Figure 7). SQR9 and ZHR0 had the most remarkable overlap among these strains, with 3,352 shared clusters, followed by FZB42, DSM7, and 168, with 3,296, 3,230, and 3,119 shared clusters, respectively. Additionally, six clusters were unique to ZHR0 (Figure 7), containing 19 proteins, 11 of which were of unknown function. At the same time, the remaining eight were involved in carotenoid biosynthetic, DNA-mediated transposition, bacteriocin biosynthesis, and antibiotic biosynthesis.

### 3.5 Detection and antifungal capacity of antimicrobial substances in ZHR0

**Figure 8:**
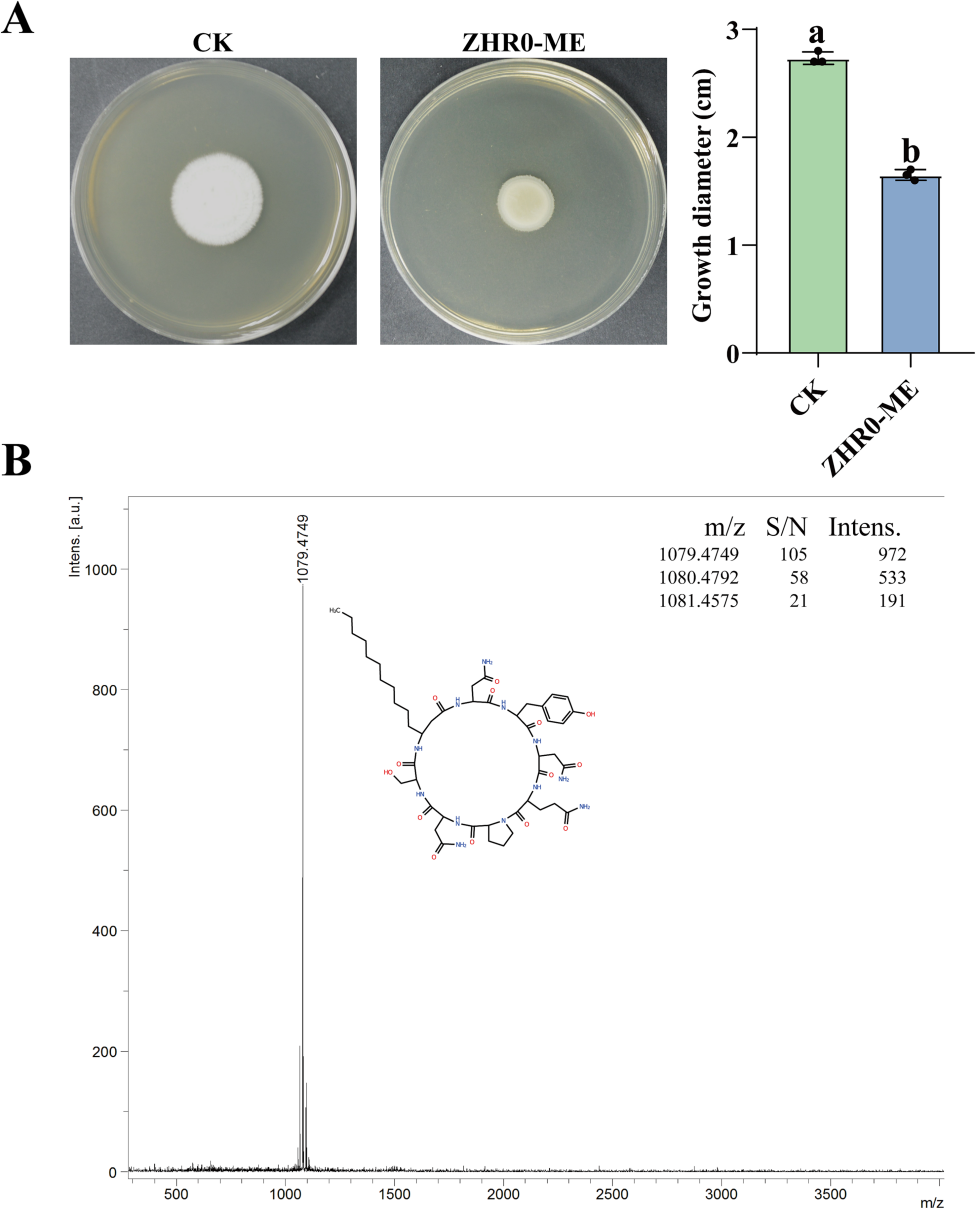
Antifungal activity of ZHR0 and LC–MS detection. **(A)** Inhibitory effect of ZHR0 methanolic extract on the growth of *Sporisorium scitamineum*, ZHR0-ME: ZHR0 methanolic extract. Error bars represent standard errors. Different lowercase letters indicated a significant difference between the two treatments according to the Student’s t-test (*p* < 0.05). **(B)** Mass spectra analysis of iturin from methanol extracts of ZHR0.

To demonstrate the antifungal capacity of ZHR0 metabolites, crude extracts of antimicrobial substances were obtained from the cell-free supernatant of ZHR0 *via* acid precipitation followed by methanol extraction. In the plate assay, the methanol extract of ZHR0 significantly inhibited the growth of *S. scitamineum*, achieving an inhibition rate of 39.61% (Figure 8A). Advanced analytical profiling was performed utilizing liquid chromatography-mass spectrometry (LC-MS) analysis to further characterize the antimicrobial substances in the methanol extract. The mass spectra of ZHR0 metabolites revealed three prominent peaks with m/z values of 1,079.4349, 1,080.4425, and 1,081.4284, respectively, with the peak at m/z 1,079.4349 exhibiting the highest intensity (Figure 8B). Based on the above results, it was preliminarily concluded that the metabolites of the ZHR0 strain contained iturin and its homologous isomers (Yu et al., 2023a).

### 3.6 Key genes potentially contributing to biocontrol in ZHR0

**Figure 9:**
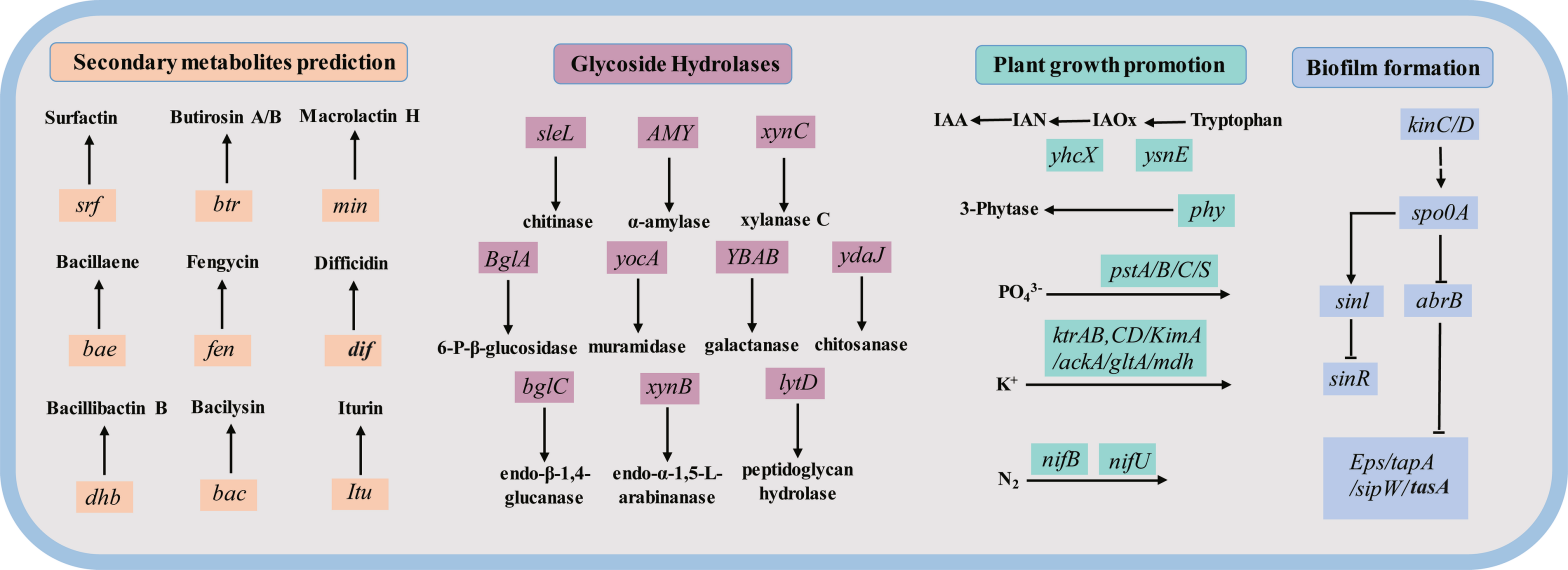
The key genes potentially participating in secondary metabolites prediction (orange color), Glycoside Hydrolase base on the CAZy database (purple color), plant growth promotion (green color), and biofilm formation (blue color) in the ZHR0 genome.

Annotation results from six databases provided significant insights into the potential roles of genes within the ZHR0 genome, particularly regarding antifungal activity, plant growth promotion, colonization, and biofilm formation (Figure 9). As previously mentioned, the ZHR0 genome contained multiple biosynthetic gene clusters responsible for synthesizing a variety of antifungal secondary metabolites (e.g., surfactin, fengycin, and iturin). Moreover, annotation through the CAZy database identified an abundance of glycoside hydrolases in the ZHR0 genome, including enzymes such as chitinase, *α*-amylase, xylanase C, 6-P-*β*-glucosidase, muramidase, galactanase, chitosanase, endo-β-1,4-glucanase, endo-β-1,5-L-arabinanase, and peptidoglycan hydrolase. These enzymes might contribute to antifungal activity and *Bacillus* colonization. In addition to antifungal properties, the ZHR0 genome also harbored numerous genes directly or indirectly involved in plant growth promotion. For example, the *ysnE* and *yhcX* genes facilitated the conversion of tryptophan to indoleacetic acid (Shao et al., 2015), while the *phy* gene encoded phytase synthesis (Rasul et al., 2021). Furthermore, genes encoding the acquisition and utilization of essential nutrients, such as PO_4_^3−^ (*pstA/B/C/S*), potassium K^+^ (*ackA*, *gltA*, and *mdh*), and nitrogen N_2_ (*nifU*, *nifB*), were also identified. A comprehensive biofilm formation pathway was identified within the ZHR0 genome, involving *kinC/D*, *spo0A*, *abrB*, *sinI*, *sinR*, and *Eps/tapA/sipW/tasA* operon (Yu et al., 2016). In general, this comprehensive genomic analysis reveals the potential of ZHR0 as a robust biological control agent with promising applications in plant growth promotion, colonization, and biofilm formation.

### 3.7 Detection of extracellular enzyme production and growth-promoting traits of strain ZHR0

**Figure 10:**
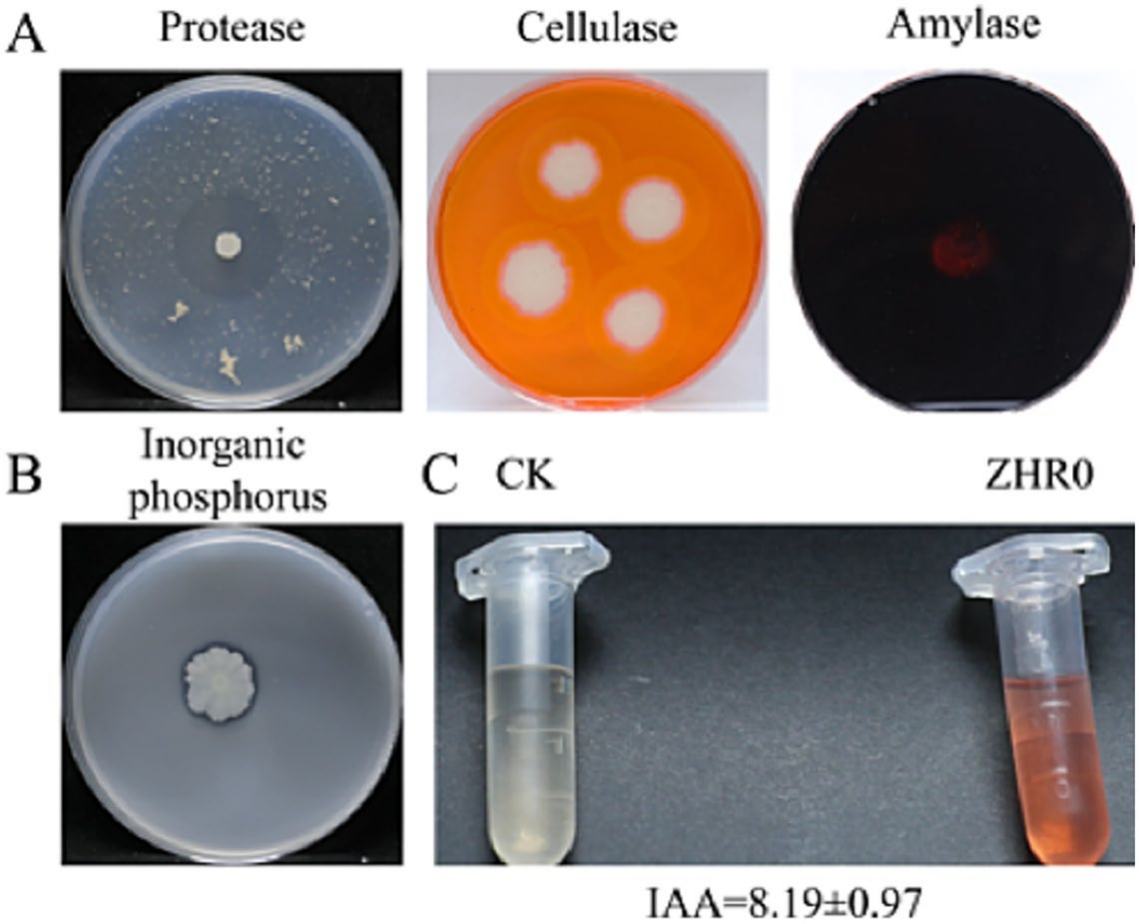
Detection of extracellular enzyme production and growth-promoting traits of *Bacillus velezensis* GSBZ09. **(A)** Extracellular enzyme activities of ZHR0, sequentially demonstrating protease, cellulase, and amylase production from left to right. **(B)** Phosphate solubilization capacity of ZHR0. **(C)** IAA production capacity by ZHR0. Data are presented as mean ± SD (*n* = 7 biological replicates).

To support genomic predictions, phenotypic assays assessed IAA production, extracellular enzyme activities (protease, cellulase, amylase), and phosphate solubilization. ZHR0 produced 8.19 ± 0.97 mg/L IAA and formed clear hydrolysis zones on all enzyme media (Figure 10), validating the functionality of biosynthetic gene clusters associated with plant-beneficial traits. These results provide empirical support for *in silico* gene function annotations.

Evans blue staining further revealed significant differences in cell integrity between control and treated *S. scitamineum* mycelia. Untreated hyphae showed only faint blue staining with intact morphology, whereas mycelia exposed to ZHR0 methanol extract exhibited extensive blue staining (Supplementary Figure S3). These observations indicated that ZHR0 methanol extract disrupted *S. scitamineum* cell membrane permeability and compromises cell wall integrity, leading to irreversible damage and growth inhibition.

## 4 Discussion

Sugarcane smut, caused by *Sporisorium scitamineum*, is one of the most devastating diseases affecting sugarcane worldwide (Wu et al., 2022b). As the demand for sustainable agricultural practices increases, BCAs are gaining recognition as environmentally friendly and cost-effective alternatives to chemical fungicides (Yu et al., 2023b). Endophytic bacteria represent a valuable source of BCAs, with *Bacillus* species being among the most promising candidates due to their broad-spectrum antifungal and antibacterial activities, which help mitigate crop losses globally (Dubey et al., 2020; Liang et al., 2021; Zhang et al., 2022; Yu et al., 2023a). In this study, we isolated and characterized an endophytic bacterial strain, *Bacillus velezensis* ZHR0, which demonstrated broad-spectrum antimicrobial activity against deuteromycetes and ascomycetes *in vitro*. In particular, ZHR0 exhibited strong antifungal activity against *S. scitamineum*. The antagonistic effects of ZHR0 included the ability to attach and erode fungal structures, thereby disrupting pathogen growth. Previous research has also highlighted the significance of *Bacillus* species in plant disease biocontrol. For instance, *B. siamensis* QN2MO-1 effectively inhibited tomato *Fusarium* wilt (Zhang et al., 2024), while *B. halotolerans* and *B. amyloliquefaciens* exhibited potent control against *Botrytis cinerea* and *Penicillium* in strawberries and loquat, respectively (Wang et al., 2021; Ye et al., 2021). Similarly, *B. velezensis* T9 effectively controlled *Apiospora arundinis*, the causal agent of *Apiospora* mold in sugarcane (Liao et al., 2023). In addition, *B. velezensis* YC89, isolated from sugarcane leaves, inhibited 78% of sugarcane red rot pathogens (Xie et al., 2023). The biocontrol efficacy of *Bacillus* species is attributed to multiple disease suppression mechanisms, including: (1) competitive exclusion of pathogenic microorganisms through rapid niche occupation (Situ et al., 2023), (2) secretion of antifungal secondary metabolites that inhibit pathogen growth (Wang et al., 2024), (3) induction of systemic resistance (ISR) and activation of host immune responses (Nguyen et al., 2022). In this study, we evaluated the *planta* antifungal activity of *B. velezensis* ZHR0 using a mixed inoculation approach, significantly reducing sugarcane smut incidence and demonstrating high disease control efficacy. Antimicrobial metabolites play pivotal roles in *Bacillus*-pathogen antagonism. For instance, iturin A derived from *Bacillus* spp. suppresses *Aspergillus niger* growth through tripartite mechanisms: disrupting cell membrane integrity, inducing oxidative stress, and interfering with central carbon metabolism (Wang et al., 2024). Notably, our study demonstrated that the methanolic extract of ZHR0 significantly inhibited the mycelial growth of *S. scitamineum* on PDA plates. This antifungal activity suggested that ZHR0-derived metabolites likely disrupted spore membrane integrity during early infection stages, suppressing fungal growth and preventing host colonization, thereby reducing sugarcane smut incidence. *Bacillus* metabolites could activate plant systemic resistance to enhance disease resistance. Iturin has been identified as a critical elicitor of this induced resistance, as evidenced by the abolished biocontrol efficacy in iturin-deficient mutants (Lam et al., 2021). This potential mechanism in ZHR0 might contribute to the significant reduction of smut incidence, which will be experimentally validated in subsequent studies.

ZHR0 showed promising control efficacy in pot experiments and a single-location field trial, broader multi-location and multi-season field studies are necessary to validate its robustness and consistency under variable agronomic conditions. Such trials are currently in the planning phase to evaluate efficacy across different sugarcane varieties and ecological zones.

Biological control agents (BCAs) and organic amendments are generally more conducive to the survival and activity of beneficial microorganisms in agricultural soils (Leal et al., 2021). Composting is an effective approach for transforming organic waste materials into stabilized products that enhance soil physical structure, nutrient availability, and microbial diversity (Sánchez et al., 2017). In the present study, *B. velezensis* ZHR0 was incorporated into a compost mixture composed of spent mushroom substrate and sugarcane bagasse to formulate a biofertilizer. This biofertilizer was subsequently applied in a field setting. BCAs delivered *via* compost-based biofertilizers have demonstrated enhanced disease suppression in various cropping systems. For example, the bioorganic fertilizer BIO2, containing *B. subtilis* N11, has been shown to effectively reduce banana wilt caused by *Fusarium oxysporum* (Zhang et al., 2011). Consistent with these findings, field application of the *B. velezensis* ZHR0-based biofertilizer in our study resulted in a significant reduction in sugarcane smut incidence, achieving a control efficacy of 43.86% (Table 2).

Whole-genome sequencing has greatly facilitated the exploration of genetic traits and functional mechanisms in *Bacillus* species, particularly those related to biocontrol and secondary metabolite biosynthesis. Since the publication of the complete genome of *Bacillus subtilis* 168 in 1997 (Kunst et al., 1997), multiple *B. velezensis* genomes have been sequenced, providing a valuable genetic framework for studying antifungal metabolite production. *B. velezensis* is known to synthesize diverse secondary metabolites that play critical roles in suppressing fungal pathogens. For example, *B. velezensis* SF305 harbors gene clusters encoding nonribosomal peptide synthetases (NRPS) and locillomycin, which contribute to both fungal inhibition and plant growth promotion (Tu et al., 2024). In the present study, genome analysis of *B. velezensis* ZHR0 revealed a repertoire of biosynthetic gene clusters (BGCs) associated with the synthesis of NRPS and polyketide synthases (PKS). These enzymes are key components in the biosynthesis of antifungal compounds (Dorrestein and Kelleher, 2006). Several of the identified BGCs shared 100% sequence similarity with well-characterized clusters in other *Bacillus* species, including those responsible for the production of macrolactin H, bacillaene, fengycin, difficidin, bacillibactin, and bacilysin, all compounds with documented antifungal activity. In particular, surfactin, fengycin, and iturin, three major classes of lipopeptides, have been extensively reported to synergistically enhance biocontrol efficacy against a variety of phytopathogens (Rahman et al., 2015).

Iturin is a well-characterized cyclic lipopeptide that exerts antifungal activity through direct interactions with fungal cell membranes. By integrating into membrane bilayers *via* sterol binding, iturin forms transmembrane pores that disrupt membrane potential and ionic gradients, ultimately leading to cytoplasmic leakage and loss of cellular homeostasis. These effects are often accompanied by oxidative stress, including increased reactive oxygen species (ROS) accumulation and lipid peroxidation, contributing to fungal cell death (Jiang et al., 2020; Zhang et al., 2025). In this study, we identified the complete iturin biosynthetic gene cluster in the *B. velezensis* ZHR0 genome, and LC-MS analysis confirmed the presence of iturin in the strain’s methanolic extract. This finding is consistent with previous reports of iturin A production by *B. amyloliquefaciens* B128, which demonstrated broad-spectrum antifungal activity (Hsieh et al., 2008). Moreover, transcriptomic data have shown that iturin A mediates its antifungal effects *via* coordinated downregulation of genes involved in membrane biogenesis, solute transport, osmoregulation, redox homeostasis, and energy metabolism, as illustrated in *Aspergillus carbonarius* (Jiang et al., 2020). These findings collectively support the multifaceted role of iturin in fungal suppression and its likely contribution to the biocontrol efficacy of strain ZHR0. Future work will include microscopy-based assays of membrane integrity and quantitative ROS measurements in *Sporisorium scitamineum* following treatment with purified iturin to validate these mechanistic pathways in this specific host-pathogen context.

Beyond its biocontrol potential, *B. velezensis* ZHR0 also harbors genetic determinants associated with plant growth promotion (PGP), underscoring its multifunctionality. The genome encodes *ysnE* and *yhcX*, which are involved in the biosynthesis of indole-3-acetic acid (IAA) from tryptophan *via* the IAM and nitrilase pathways, respectively. IAA is a key phytohormone that regulates root development and plant cell differentiation (Keswani et al., 2020). ZHR0 also encodes a diverse array of glycoside hydrolases, such as chitosanase, chitinases, endo-*β*-1,4-glucanase, 6-phospho-β-glucosidase, endo-*α*-1,5-L-arabinanase, and peptidoglycan hydrolase, which contribute to both pathogen degradation and effective rhizosphere colonization (Leal and SÃ¡-Nogueira, 2004; Reinhold-Hurek et al., 2006; Shafi et al., 2017).

In terms of nutrient acquisition, ZHR0 possesses multiple genes involved in phosphate solubilization (*pstA/B/C/S*), inorganic phosphorus metabolism (*3-phytase*), potassium solubilization (*ackA*, *gltA*, *mdh*), and potassium transport (*ktrAB*, *ktrCD*, *kimA*), facilitating the mobilization and uptake of essential nutrients (Qi et al., 1997; Adams et al., 2008; Richardson et al., 2011; Gundlach et al., 2017; Chen et al., 2022). The presence of *nifB*, *nifU*, and *nifS* genes also suggests a capacity for biological nitrogen fixation, potentially reducing the need for synthetic nitrogen inputs (Satyanarayana et al., 2018). Furthermore, ZHR0 harbors key genes involved in biofilm formation, which are critical for environmental persistence, root colonization, and stress tolerance (Yu et al., 2016). Phenotypic assays confirmed ZHR0’s functional traits, including extracellular protease, cellulase, and amylase activity, as well as phosphate solubilization and IAA production. These features are consistent with the growth-promoting profile observed in related strains, such as *B. velezensis* GSBZ09, which has demonstrated similar enzymatic activities and growth enhancement effects in grapevine cultivation systems (Yin et al., 2022). Altogether, the genomic and functional data support the classification of ZHR0 as a multifunctional plant-beneficial bacterium with dual capabilities in biocontrol and plant growth promotion. This makes it a promising candidate for integration into sustainable agriculture and biofertilizer development strategies.

## 5 Conclusion

In this study, we isolated and characterized *B. velezensis* ZHR0, an endophytic strain exhibiting strong antifungal activity against sugarcane smut. ZHR0 demonstrated broad-spectrum antifungal effects *in vitro* and significantly reduced disease incidence under greenhouse conditions. Field application of a ZHR0-based biofertilizer further confirmed its effectiveness in controlling sugarcane smut. Whole-genome analysis identified multiple biosynthetic gene clusters responsible for the production of secondary metabolites, including iturin, which was experimentally validated through LC-MS. In addition to its biocontrol properties, ZHR0 harbors multiple plant growth-promoting traits. Collectively, these findings highlight the potential of *B. velezensis* ZHR0 as a dual-function biocontrol agent and biofertilizer for sustainable sugarcane cultivation.

## Data Availability

The data presented in this study are deposited in the NCBI GenBank repository under accession number PV565712 (https://www.ncbi.nlm.nih.gov/genbank/).

## Footnotes

^1^ https://jspecies.ribohost.com/jspeciesws/#home

^2^ https://www.chiplot.online/

^3^ https://orthovenn3.bioinfotoolkits.net/

## Glossary

BCAs: Biological control agents
PCR: Performed polymerase chain reaction
ANI: Average nucleotide identity
BGC: Biosynthetic gene clusters
GO: Gene Ontology
KEGG: Kyoto Encyclopedia of Genes and Genomes
LC-MS: Liquid chromatography-mass spectrometry
NRPS: Nonribosomal peptides synthetase
IAA: Indole-3-acetic acid
IAM: Indole-3-acetamide
IPyA: Indole-3-pyruvic acid
TAM: Tryptamine
IAN: Indole-3-acetonitrile
TSO: Tryptophan side-chain oxidase
Pst: Phosphate transport system
LB: Luria-Bertani
CCTCC: China Center for Type Culture Collection
PDA: Potato dextrose agar
ddH2O: Double-distilled water
OLC: Overlap-layout-consensus
CFS: Cell-free supernatant
ME: Methanol crude extract
UHPLC: Ultra-high-performance liquid chromatography
HESI: Heated electrospray
