# Supplementary figures and images for: Genomic and metabolomic insights into the biocontrol potential of Bacillus velezensis ZHR0 against sugarcane smut
Source: Front Microbiol. 2025 May 13;16:1582763. doi: 10.3389/fmicb.2025.1582763 (PMC12106456; doi:10.3389/fmicb.2025.1582763)

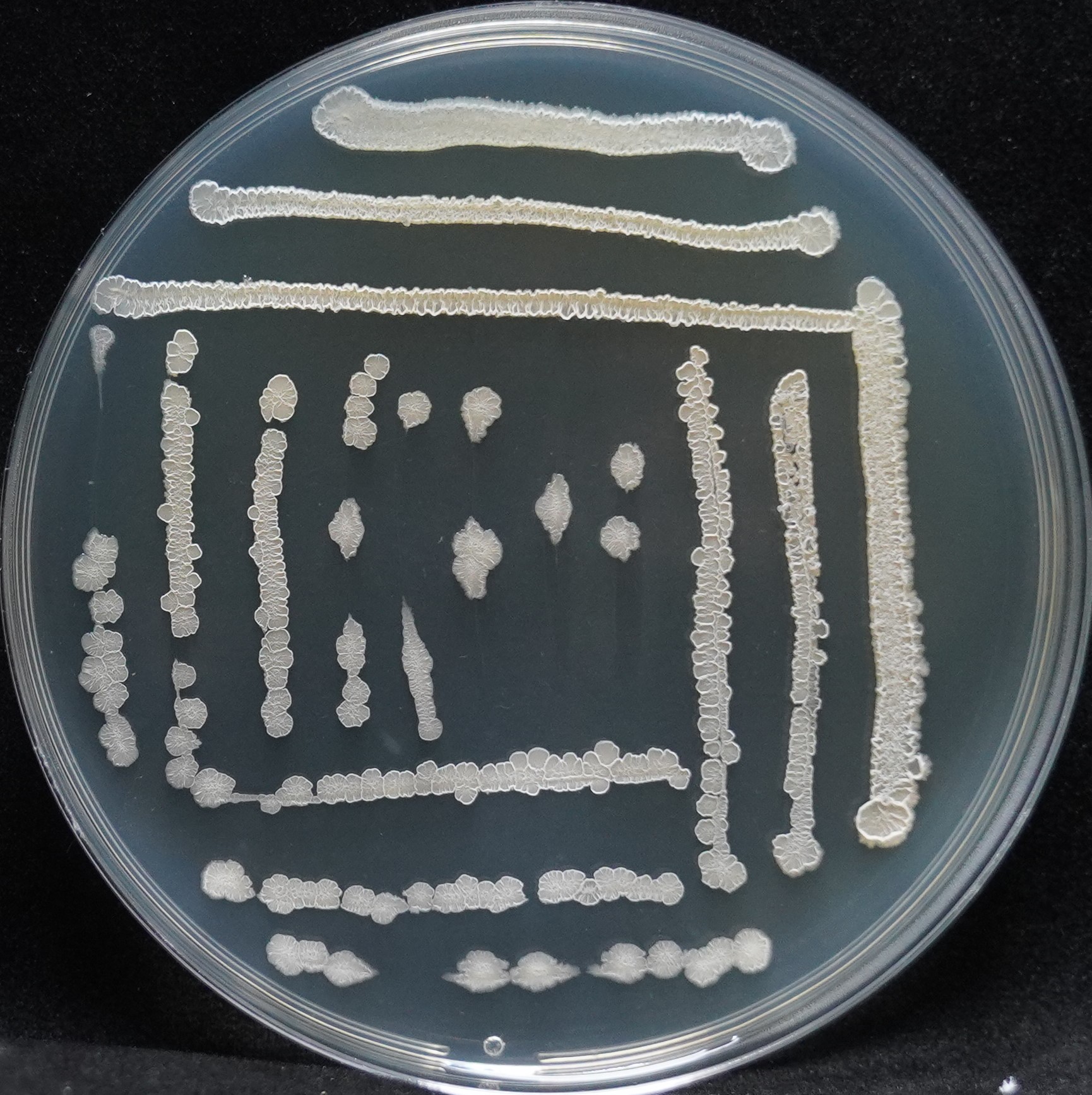

Supplement: Supplementary file 1 [file Image_1.JPEG]

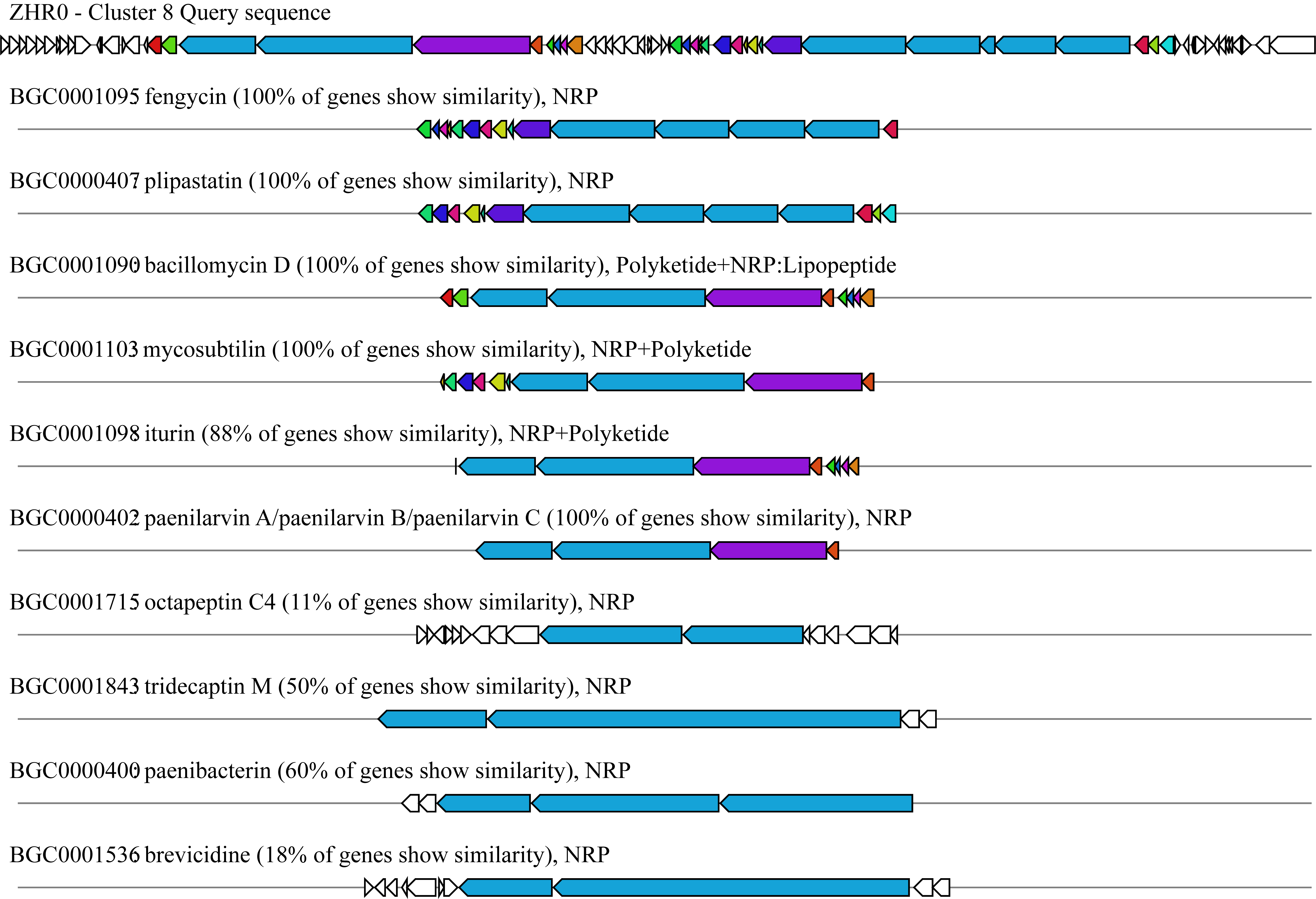

Supplement: Supplementary file 2 [file Image_2.TIF]

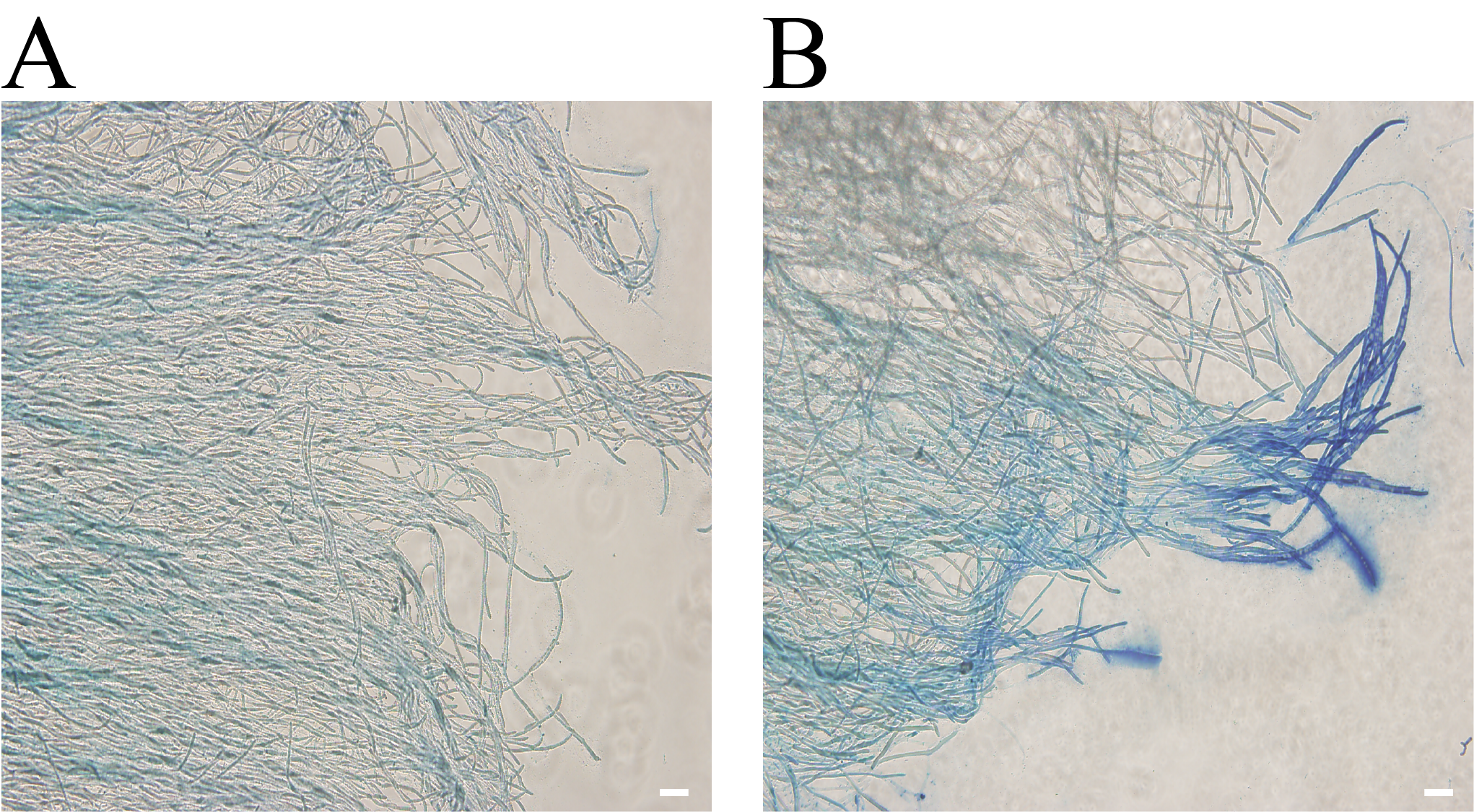

Supplement: Supplementary file 3 [file Image_3.TIF]
