# Supplementary material for: Genomic and metabolomic insights into the biocontrol potential of Bacillus velezensis ZHR0 against sugarcane smut
Source: Front Microbiol. 2025 May 13;16:1582763. doi: 10.3389/fmicb.2025.1582763 (PMC12106456; doi:10.3389/fmicb.2025.1582763)
Supplement: Supplementary file 4 [file Table_1.DOCX]

**Table S1** Number of genes annotated in each database

| Values | Gene Number | Percentage |
| --- | --- | --- |
| Total | 4,150 | 100% |
| Nt-Annotated | 4,132 | 99.57% |
| Nr-Annotated | 4,128 | 99.47% |
| Swissprot-Annotated | 3,616 | 87.13% |
| COG-Annotated | 3,005 | 72.41% |
| GO-Annotated | 2,955 | 71.20% |
| KEGG-Annotated | 2,176 | 52.43% |
| Overall | 4,149 | 99.98% |
